# Claudins in ovarian cancer: emerging biomarkers and therapeutic targets

**DOI:** 10.1080/21688370.2025.2539026

**Published:** 2025-08-13

**Authors:** Lubna Therachiyil, Ajaz A. Bhat, Shahab Uddin

**Affiliations:** a Translational Research Institute, Hamad Medical Corporation, Doha, Qatar; b Metabolic and Mendelian Disorders Clinical Research Program, Precision OMICs Research & Translational Science, Sidra Medicine, Doha, Qatar; c Dermatology Institute, Academic Health System, Hamad Medical Corporation, Doha, Qatar; d Laboratory of Animal Center, Qatar University, Doha, Qatar; e Department of Biosciences, Integral University, Lucknow, India

**Keywords:** Biomarker, Claudins, ovarian cancer, therapeutic target, tight junction

## Abstract

Tight junctions (TJ) comprise protein complexes that help with the movement of ions and molecules through the paracellular pathway, thus maintaining both epithelial and endothelial integrity. The TJ proteins are diverse and include claudins, occludins, tricellulins, cingulins, and junctional adhesion molecules (JAM). Claudins are transmembrane proteins that serve as critical components of TJs in epithelial and endothelial cells. The human genome comprises 23 claudin genes, with 27 transmembrane domains recognized in mammals. Ovarian cancer (OC) is the most lethal form of all gynecologic malignancies worldwide, characterized by poor prognosis and a recurrence rate of up to 75%. In OC, several claudins are overexpressed relative to normal ovarian tissue. These elevated expression observed among OC subtypes indicates their potential utility as diagnostic biomarkers. Claudins represent potential targets for innovative therapeutic strategies. Though their exact involvement in OC is still not well understood, they are believed to be crucial for cancer invasion and therapy resistance. Recent studies show that claudins are involved in the EMT pathway and ERK, enlightening the effect of claudins in drug resistance. Clostridium perfringens enterotoxin (CPE) demonstrates potential as a therapy targeting claudins, specifically claudin-3 and −4, which serve as receptors for this toxin. Despite these advancements, challenges remain in comprehensively understanding claudin functions and in the development of effective claudin-targeted therapies. This review consolidates existing knowledge regarding claudins in OC, focusing on their expression patterns, biological functions, diagnostic and prognostic significance, and therapeutic implications. A thorough understanding of claudins in OC establishes a basis for enhancing diagnostic, predictive, and therapeutic approaches, which may result in improved therapy outcomes.

## Introduction

Ovarian Cancer (OC) is the most lethal form of all gynecologic malignancies worldwide, characterized by poor prognosis and a recurrence rate of up to 75%.^1^ It is characterized by nonspecific early symptoms that make two-thirds of patients diagnosed only at late stages (II-IV).^2^ A proposed lifetime risk of OC in a woman is 1 in 75, and the death rate is 1 in 10.^5^ Epidemiologically, the occurrence of OC is highly observed in developed countries, including North America and Europe.^3^ Both benign and malignant OCs originate from any of the following three cell types: stromal cells, epithelial cells, and germ cells. More than 90% of the cases of OC in developed countries are found to be of epithelial origin.^4^ Epithelial OC (EOC) has several histotypes based on their origin, pathogenesis, gene expression, molecular alterations, and prognosis. Malignant OC, which is also known to be carcinomas, include five main histotypes: high-grade serous, endometrioid, mucinous, clear cell and low-grade serous.^5–7^ Most of the tumors are found to originate in other gynecological tissues and secondarily invade the ovaries. Family history is found to be one of the leading risk factors associated with OC, where the relative risk is estimated to be 3 to 7 fold with a first-degree relative affected with OC.^8^ Other significant risk factors are observed to be smoking, several gynecological situations such as Polycystic Ovary Syndrome, endometriosis, pelvic inflammatory disease (PID), and use of oral contraceptives^9^ infertility, ovulation stimulating drugs for subfertility,^10^ obesity^11^ nutrition, exercise, and physical activity.^3^ Prolonged lactation is found to have a protective effect against OC, as suggested by some researchers.^12,13^ A relation between hormone replacement therapy and the occurrence of OC is identified.^14^ Studies show that the BRCA1/2 gene is the most critical and common gene predisposing to OC associated with high-grade serous histology.^15^ Other genes include BRIP1, RAD51C, and RAD51D, MSH6, MSH2, and MLH1 which are mismatch DNA repair genes.^16,17^

## Tight junctions

Tight junctions (TJ) comprise protein complexes that help with the movement of ions and molecules through the paracellular pathway, thus maintaining both epithelial and endothelial integrity.^18^ They also aid in the movement of proteins and lipids between the apical and the basolateral domains of the plasma membrane.^19^ Disintegration of tight junctions is known to increase the risk of many diseases, including cancer.^20^ The TJ proteins are diverse and include claudins, occludins, tricellulins, cingulins, and junctional adhesion molecules (JAM). They are found to have a fence function by forming an apical/basolateral intramembrane diffusion barrier, which prevents the mixing of membrane proteins. Additionally, they have a gate function by controlling the breadth and selectivity of diffusion along the paracellular pathway. Involvement of TJ proteins in cell proliferation has also been identified. Moreover, they are also involved in gene expression by modifying signaling pathways or by confiscating transcription factors.^21^

## Claudins-structure and functions

Claudins, the principal components of the tight junctions^22^ are the most important proteins in forming the paracellular barriers and signal transduction.^23^ They belong to the PMP-22 (peripheral myelin protein 22), a protein necessary for peripheral nerve myelin formation. Approximately 27 members of the claudin family have been identified in mammalian cells till now, conferring various expression profiles depending on location and cell type.^24^ The mRNAs encoding mammalian claudins are assembled between exons 1 and 6. For some of the mRNAs encoding claudins, splice variants have been observed conferring isoform existence in claudins with differential expressions and ability to co-polymerize into heteropolymers by homophilic and heterophilic interactions.^25^

**Figure 1.**
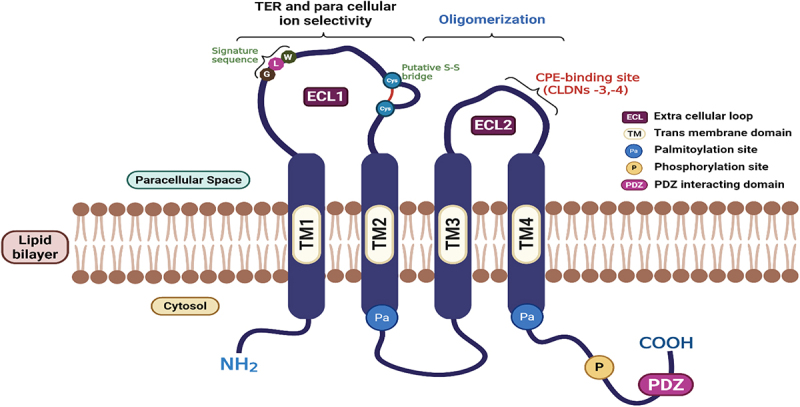
Schematic representation of claudin structure.

Most claudins possess between 207 and 305 amino acids and have a molecular weight ranging from 20–34 kDa. They have four transmembrane helices, an amino and carboxyl cytoplasmic domains and two extracellular loops (ECL 1, larger and ECL 2, smaller); the loop containing charged amino acids plays a central role in paracellular ion selectivity, and the other loop is essential for localizing the claudin to tight junctions (Figure 1). All except claudin-12 possess a carboxyl-terminal tail that encompasses a PDZ-domain-binding motif, which facilitates claudins to interact directly with cytoplasmic tight junction-associated scaffolding proteins.^26^ The carboxy-terminal end assists the localization of claudins to the TJ complex.^26^

Claudin members share a similar structure. Each claudin molecule has four transmembrane helices, an amino and carboxyl cytoplasmic domains, and two extracellular loops (ECL 1, larger and ECL 2, smaller); the loop containing charged amino acids plays a central role in paracellular ion selectivity, and the other loop is important for localizing the claudin to tight junctions. All except claudin-12, all possess carboxyl-terminal tails that encompass a PDZ-domain-binding motif, which facilitates interaction of claudins directly with cytoplasmic tight junction-associated scaffolding proteins. The carboxy terminal also has several key sites for post-translational modifications, such as phosphorylation and palmitoylation. Created in https://BioRender.com.

Most tissues express multiple claudins^27^ and they are classified into two types based on their functions as barrier-forming claudins (claudin-1,-3,-4, and −5) and pore-forming claudins (claudin-2, −7,-10, and −16).^27^ Over expression of claudins is found to cause increased permeability to different ions and can affect trans-epithelial resistance (TER).^28^ Claudins are also separated into two diverse groups based on the homology shared by them in terms of the amino acid sequences. They are termed as classic sharing higher homology (claudin-1 to −10, −14, −15, −17, −19) and non-classic sharing lower homology (claudin-11, −12, −13, −16, −18, −20 through −24).^29^ Their expression in various cells is found to be regulated by the Epidermal Growth Factor signaling (EGFR), MEK/ERK or PI3K/AKT,^30^ and NOTCH pathways.^31^

## Claudins and cancer

Evidences show that disruption of cell-to-cell adhesion is observed and is an essential path in cellular transformation and tumor metastasis.^32^ proper cell-to-cell and cell-to-extracellular matrix interactions are crucial for the typical functioning of both tissues and organs. Similarly, the claudins are pretty likely to have a central role in tumorigenesis. Claudins are observed to be both tumor-promoting as well as tumor-suppressing depending on their functional specificity, cancer types, and subtypes.^33^ In melanoma, thyroid, colorectal, ovarian, hepatocellular, squamous, and pancreatic cancers, claudins are tumor promoters, whereas in lung and prostate, they are tumor suppressors.^26^

Several claudin members are found to be either deregulated (up- or down-regulated) or dislocated in several cancers, including ovarian,^34–37^ breast,^38,39^ colorectal,^40,41^ gastric adenoma,^42–45^ hepatocellular,^46,47^ pancreatic,^48,49^ oral squamous cell,^50,51^ melanoma^52,53^Hypopharyngeal squamous cell and thyroid cancers.^54–57^ Of all the claudins studied till now, claudin-1, −3, −4, −6, −7, −10, −16, and −18.2 are the most often dysregulated ones observed in cancer, both at transcriptional and post-transcriptional levels.^58^ Claudin shows a complex, organ-dependent expression in terms of normal cells, benign tumors, and malignant cancers.^59^ In breast and prostate cancer, claudins −1 and −7 are down-regulated, but claudins −3 and −4 are up-regulated.^26^ In OC, claudins −1 through −7 and −16 are found to be deregulated, out of which claudins-3, −4, −7, and −16 are up-regulated.^27^ In colorectal cancer, claudin-1 is up-regulated and −7 is either up-regulated^60^ or down-regulated.^61,62^

## Claudins and cancer drug resistance

Emergence of drug resistance is a major factor in cancer cells that reduces drug efficacy.^63^ Although numerous molecular mechanisms underlying drug resistance have been studied, the existence of cancer stem cells, also known as tumor initiating cells, is found to be a major cause. The Epithelial to Mesenchymal Transition (EMT) pathway is associated with the initiation of cancer stemness, thereby inducing resistance.^64^ Recent studies show that claudins are involved in the EMT pathway, which enlightens the effect of claudins in drug resistance. The WNT/β Catenin pathway is also important in attaining stemness in cancer cells. Recent studies show that claudin-1 and −2 gene transcription is initiated by the WNT/β-Catenin pathway.^65^ Reports suggest that depletion of claudin-3 in human non-small cell lung cancer cell lines reduces the formation of spheres and tumor formation and increases sensitivity to cisplatin.^66^ A recent article shows that Claudin-2 promotes self-renewal of human colorectal cancer stem-like cells.^67^

## Biological functions of claudins in OC

Differential expression of several claudins are observed in OC. More specifically, claudins are found to be overexpressed in OC.^68^ They tend to serve different functions in a claudin-dependent manner, including both tumor promotion and tumor suppression. Major claudins dysregulated in OC are found to be claudin-1,-3, −4, −5, 6, 7 and −16.^27^ Given the higher expression of claudins in OC in comparison to normal ovarian cells, their pivotal roles in response to chemotherapeutic drugs and tight junction (TJ) formation, as well as their importance in tumorigenesis, this review seeks to explore the potential applications of claudins in OC management, both as biomarkers and as therapeutic targets. This section highlights the current available insights into understanding how claudins modulate OC, bridging the roles with their promising significance as potential drivers of carcinogenesis. Comprehending these functional involvements can lay a strong foundation for leveraging them in both prognostic, diagnostic and moreover therapeutic strategies. Below we discuss in detail, the role and functional characteristics of different claudin members in OC. Table 1 illustrates the overexpressed claudin members and their potential significant roles in OC.

**Table 1.** Claudins in OC and their functional significance. Several claudins are found to be overexpressed in various histotypes of OC. Many claudins are also reported to be potential therapeutic targets for OC treatment and management.

Claudin	Associated OC type	Significance in OC	Reference
Claudin-1	Serous, endometrioid	Cancer growth and invasion, chemoresistance and favors poor survival	^73,74^
Claudin-3	Serous, endometrioid	Induces EMT and metastasis, shortens overall patient survival	^68,78,79^
Claudin-4	Serous, mucinous	Promotes tumor aggressiveness, cancer cell survival, metastasis, and chemoresistance	^36,86,97^
Claudin-5	Serous, mucinous and endometrioid	Worsen cancer-specific survival and OS	^97^
Claudin-6	Serous	Promotes invasion and metastasis	^105^
Claudin-7	All epithelial and serous	Promotes chemoresistance and worsens progression-free survival, invasion and metastasis	^109,111,124^
Claudin-10	Epithelial	Shorten overall patient survival	^114^
Claudin-16	Epithelial, Clear cell	Possible cancer initiation	^118^
Claudin-18.2	primary and advanced mucinous	Possible therapeutic target	^120^

### Claudin-1 (CLDN1)

CLDN1 is located on chromosome 3q28 and has one CpG island, which is located at the promoter region. It is illustrated as the most dysregulated member in cancer, and its expression in OC is extensively studied in relation to tumor growth as well as progression.^69^ The role of CLDN1 in EMT and the ERK pathway is also established.^70^ In OC, the overexpression of CLDN1 is established, which is directly correlated with lowering cell differentiation and promoting cancer growth and invasion.^71^ Visco et al reports that epigenetic factors, especially DNA methylation, modulate CLDN1 expression. Moreover, using short-term and long-term survivor data analysis, they found a negative correlation between CLDN1 expression and DNA methylation in short-term survivors and not in others. Their data suggest that tumor migration and invasiveness are diminished when CLDN1 is inhibited both chemically and genetically.

Furthermore, the study shows that claudin knockdown increased drug sensitivity in SKOV8 and CAOV2 cell lines toward paclitaxel and SKOV3 toward carboplatin. Furthermore, they found that CLDN1 knockdown resulted in suppression of stemness markers CD44 and CD133. Their data also shows that mice injected with SKOV3 cells with knockdown CLDN1 (ShRNA- CLDN1) gene inhibited tumor growth in mice upon treatment with carboplatin in comparison to mice treated with SKOV3 transduced with off-target shRNA control.^72^ Kleinberg et al reports that CLDN1 is overexpressed in ovarian carcinoma and for patients with post-chemotherapy effusions, it is associated with poor survival.^73^ CLDN1 expression was found to be correlated with peritoneal implants as well as micropapillary pattern in OC that was highly associated with poor prognosis.^74^

### Claudin-3 (CLDN3)

CLDN3 is involved in the TJ formation that regulates intercellular permeability, whose expression has been found to be deregulated and associated with tumor progression in various tumors.^75^ Hough et al., using serial analysis of gene expression (SAGE) from various ovarian cell lines and tissues, including primary cancers, ovarian surface epithelial cells, and cystadenoma cells, found CLDN3 to be one of the most highly up-regulated genes in OC.^76^ This was also reported by others in primary ovarian tumors, as ;measured by transepithelial electrical resistance; however, not in ovarian cystadenomas, implying that the expression of these proteins is linked with malignancy.^77^

In a study using 84 serous adenocarcinomas, CLDN3 expression was significantly correlated with shorter patient survival, as proved by Kaplan-Meier analysis.^78^ Moreover, the study also showed that the expression of CLDN3 is an independent prognostic factor predicting patient disease outcome through multivariable COX regression analysis. CLDN3 is also found to be a molecular marker for poor overall survival (OS), wherein the study included univariate analysis for 57 patients with disease recurrence post-chemotherapy effusions.^79^ Furthermore, Claudin-3 was found to be an independent predictor of poor overall survival for patients with post-chemotherapy effusions (*p* = .012).^73^ This group carried out an immunohistochemical staining of 218 effusions and 245 primary and metastatic tumors, confirming the overexpression of CLDN3 in OC effusions that was correlated to shorter OS, as investigated by univariate survival analysis.^73^

### Claudin-4 (CLDN4)

CLDN4 is overexpressed in most EOC, where it regulates chemoresistance by modulating genomic instability, thereby affecting nuclear and cell cycle remodeling.^80,81^ Agarwal et al. showed that both CLDNs 3 and 4 were overexpressed in OC cell lines, increased cell survival, and promoted cancer metastasis by enhancing MMP-2 activity.^36^ Honda et al. has shown that the expression of CLDN4 as well as CLDN3 are regulated by DNA methylation and histone deacetylation.^82,83^ In OC, loss of repressive histone methylations, including H3K27me3 and H4K20me3, is associated with increased expression of CLDN3 and CLDN4.^84^ Knockdown of CLDN3 and CLDN4 is shown to increase cisplatin resistance in both in ;vitro (human ovarian carcinoma 2008 cells) and in ;vivo xenograft models, wherein the net accumulation of the drug as well as the drug-DNA adduct levels were reduced in the knocked-down cells.^85^ A study aimed at determining the presence of CLDN4 in OC tissues in relation to platinum compounds resistance found that CLDN4 overexpression was observed in advanced stages of the papillary serous subtypes and is reportedly associated with tumor aggressiveness.^86^ They also show that overexpression of CLDN4 is associated with therapy resistance. Moreover, a good response to first-line chemotherapy was observed in patients with low CLDN4 expression. This study suggests CLDN4 to be a predictive marker for poor therapy response in advanced OC. Yoshida et al. reports that CLDN4 expression was associated with significantly poorer prognosis and also contributes to platinum resistance in OC. Here, they analyzed the CLDN4 expression in 43 OC patients that has undergone chemotherapy with cisplatin. This could be due to the overall strengthening of the barrier functioning that concurrently reduces the cellular accumulation of cisplatin. This suggestion is strengthened by their further study using CLDN4 siRNA that resulted in increased accumulation of fluorescent labeled cisplatin molecules in OVCAR3 and CaOV3 cell lines. They also suggests CLDN4 to be a potential therapeutic target in the treatment of platinum-resistant tumors.^87^

Fuente et al., in their study, assessed the effect of CLDN4 overexpression on progression-free survival and OS in OC patients using risk ratios and the Pearson χ^2^ ;test. They found that out of 132 patients who obtained platinum-based treatment, a patient with higher CLDN4 expression had a 1.2 times higher risk (95% CI = 0.7–2.0, ;*p* = 0.3) of being resistant to therapy in comparison to patients with lower expression of this protein, suggesting a prognostic value for CLDN4 in OC.^88^ Targeting CLDN3 and CLDN4 has been proposed as a chemotherapeutic strategy in OC.^89^ A study from Santin et ;al. carried out quantitative reverse transcription (qRT)-PCR analysis, showing a significantly higher expression of both CLDN3 and CLDN4 in established human ovarian tumors.^90^ Moreover, they also report that sustained in vitro culturing of OVCAR-3 and CaOV-3 cell lines resulted in a significant decrease in the expression of claudins in comparison to primary ovarian tumors.^90^ Another study has shown that upregulation of CLDN4 in cell lines such as OVCAR4 and OVCAR8 was linked to reduced apoptotic response to paclitaxel treatment as assessed by caspase-3 and Annexin V binding. They suggest that disrupting CLDN4 with claudin mimic peptide (CMP) could restore apoptotic response.^91^

### Claudin-5 (CLDN5)

CLDN5 is mostly expressed in endothelial cells, and it is crucial for the formation of the blood-brain barrier.^92^ A significantly high expression of claudins is observed in vascular endothelial cells and in endothelial tumors.^93–95^ CLDN5 overexpression was observed in malignant epithelial tumors such as serous cystadenocarcinomas, mucinous cystadenocarcinomas, and endometrioid adenocarcinomas. This study assessed the expression of claudins −1, −4, −5, and −7 in 60 different types of ovarian lesions, mainly consisting of ovarian neoplasms and found an enhanced expression of CLDN5, mainly in endothelial cells of ovarian epithelial tumors, and not in adenomatoid tumors which matches with their non-endothelial nature.^96^ A study by Turunen et al. investigated the link between CLDN5 expression and OC behavior. For this, they analyzed the expression of CLDN5 across 85 serous OC tissue samples using immunohistochemical staining. They report that overexpression of CLDN5 was associated with advanced high-grade (*p* = 0.016) and advanced stage (*p* = 0.022), cancer-specific survival, and OS in carcinomas. Moreover, a correlation between CLDN5 expression and cancer-specific survival (*p* = 0.032) as well as OS (*p* = 0.026) was identified. After 5 years, around 25–30% of claudin-5-positive patients survived, whereas it was around 60% in the case of CLDN5-negative patients.^97^

In a study aimed at identifying the mechanisms of ascites formation ;in OC patients, CLDN5 was found to be present in the peritoneal vessels of patients, with a weaker expression compared to the vessels of control samples. This study concluded that VEGF induces ascites in OC patients due to elevated peritoneal permeability, attributed to the downregulation of CLDN5 in the peritoneal endothelium.^98^

### Claudin-6 (CLDN6)

CLDN6 is a membrane protein present on the surface of various solid tumors, including ovarian, testicular, and endometrial cancers, with no detectable expression in normal adult tissues at both transcriptional and protein levels.^99–101^ It is considered to be a potential therapeutic target for antibody-based cancer immunotherapies. Moreover, due to its tumor-specific cell surface expression, CLDN6 is also a possible target for cancer vaccines. Antibodies against CLDN6 have already been engineered as bi-specific single-chain antibody^102^ as well as anti-CLDN6 antibody-drug conjugates for targeted immunotherapy.^103^ A previous study showed that CLDN6 expression was observed in 69.4% of ovarian carcinomas and 34.6% of ovarian serous adenomas as analyzed by IHC.^104^ This study found that CLDN6 was overexpressed in ovarian papillary serous carcinomas, and this could be correlated to increased clinical stage and metastasis. Moreover, the study suggests an important modulating role of CLDN6 in the invasion and metastasis of OC.^104^ Matsuzaki et al. investigated the spontaneously induced T-cell response against CLDN6 in OC patients, characterizing CLDN6-specific CD8+ ;and CD4+ ;T cells and identifying novel CLDN6-derived short peptides that bind to HLA-A *02:01 (A2) and -DRB1 × 04:04 (DR4). They identified TCR genes specific to CLDN6 from both CD8^+^ ;and CD4^+^ ;T cells and demonstrated specific reactivity to CLDN6^+^ ;cancer cells by TCR-transduced T cells. ;They showed that CD8+ and CD4+ T-cell responses against CLDN6 in cancer patients generate CLDN6-specific TCR-transduced T cells. Given the higher expression rate of CLDN6 on cancer cells, they suggest CLDN6 to be a promising target for TCR-engineered T-cell therapy in OC patients.^105^

### Claudin-7 (CLDN7)

CLDN7 is located at the apical TJ and basolateral side of intestinal epithelial cells.^106^ Functionally, CLDN7 plays crucial roles in several molecular signaling pathways associated with carcinogenesis, cancer progression, and cell cycle.^107^ It is found to be overexpressed in OC.^50,108,109^ In EOC, Dahiya et al. investigated the expression profile and functional characteristics of CLDN7. They studied a total of 95 OC tissue samples with varying histotypes for the constitutive expression of CLDN7 and found it to be up-regulated in all of the samples. IHC analysis and immunoblotting results from them suggest that CLDN7 is overexpressed in EOC cells. Moreover, SiRNA-mediated knockdown of CLDN7 in OC cells resulted in alterations to genes involved in various molecular functions such as cell cycle, cellular growth, proliferation, apoptosis, and cell migration. Furthermore, their study also shows that both invading and migratory properties of OC were altered with respect to knockdown or overexpression of CLDN7.^107^

Tassi et al. quantified and compared the expression of CLDN7 in 110 patients with different histologic types of EOC via RT-PCR and found it to be overexpressed in both primary and metastatic EOCs compared to normal human ovarian surface epithelium cell lines. Their immunoblot results also showed a similar result. They found an overexpression of CLDN7 not only in all main histologic types of EOC but also in single neoplastic cells disseminated in the peritoneal cavity and pleural effusions. Reports from this group suggest CLDN7 to be a potential novel diagnostic marker and possibly the right candidate for antibody-mediated localized therapies of OC.^110^ Reports also indicate a correlation between CLDN7 and shorter progression-free survival (PFS) of OC patients (*p* = 0.005) and resistance to platinum-based chemotherapy (*p* = 0.024). Moreover, siRNA of CLDN7 resulted in significant enhancement of the sensitivity of EOC cells 2774 and HeyA8 cells to cisplatin treatment.^111^ In high-grade serous ovarian carcinoma (HGSOC) patients, lower expression of CLDN7 was found to be a predictor of distant metastasis, mainly by the hematogenous route (*p* = 0.025).^112^

### Claudin-10 (CLDN10)

CLDN10 appears in two major isoforms, claudin-10a and claudin-10b, constituting paracellular anion or cation channels, respectively.^113^ CLDN10 expression was found to be significantly higher in high-grade serous ovarian carcinoma (HGSOC) effusions and surgical specimens compared to mesothelioma and is suggested to be a candidate prognostic marker for HGSOC.^114^ Li et al. conducted a study to identify novel prognostic markers for patients with OC, focusing on estimating tumor metastasis or recurrence. They found that CLDN10 acts as a novel biomarker for OC prognosis. This group did a comprehensive analysis of transcriptome data from various biorepositories in order to identify potential biomarkers. They report that CLDN10 may influence OC progression via transforming growth factor-β (TGF-β)- or WNT/β-catenin-induced EMT. Their study highlights the importance of Claudin 10 as a predictive marker for patient outcome and suggests potential therapeutic strategies for OC patients.^115^ CLDN10 is found to be a glandular epithelial marker in the chicken model, as in human EOC, as confirmed by reverse transcription-polymerase chain reaction (RT-PCR) analysis. The group performed in situ hybridization to further confirm the localization of CLDN10 mRNA in both normal and cancerous ovaries, and proposed CLDN10 to be a prognostic marker in OC.^116^ Likewise, Davidson et al. reported CLDN10 to be a new candidate prognostic marker in metastatic HGSOC. They found that CLDN10 expression was higher in HGSOC in comparison to mesothelioma, and it could be a molecular marker for enhanced aggressiveness of cancer.^114^ Moreover, the study also discusses CLDN10 to be significantly associated with shorter OS (*p* = 0.036) and PFS (*p* = 0.045) and confirms it to be ;an independent prognostic factor of OS in multivariate analysis.^114^

### Claudin-16 (CLDN16)

CLDN16 is observed to be frequently up-regulated in EOC of all subtypes.^117^ In silico analysis of CLDN 16 expression profiling was conducted using data extracted from the GENT2 and GEP1A2 platforms. As per the data, though CLDN16 is overexpressed, it was not directly related to tumor stage, drug response to cisplatin, or survival rate.^117^ The elevated expression of CLDN16 is found to be modulated by PI3K and PKC pathways.^117^ Paes et al. propose that overexpression of CLDN16 in the cytoplasm could possibly initiate malignancy thus playing a critical role in ovarian carcinogenesis. They also suggest CLDN16 to be an interesting and novel target for OC.^118^ CLDN16 signifies an exceptional protein in OC, where its overexpression suggests diagnostic and prognostic implications.

### Claudin-18.2 (CLDN18.2)

**Figure 2.**
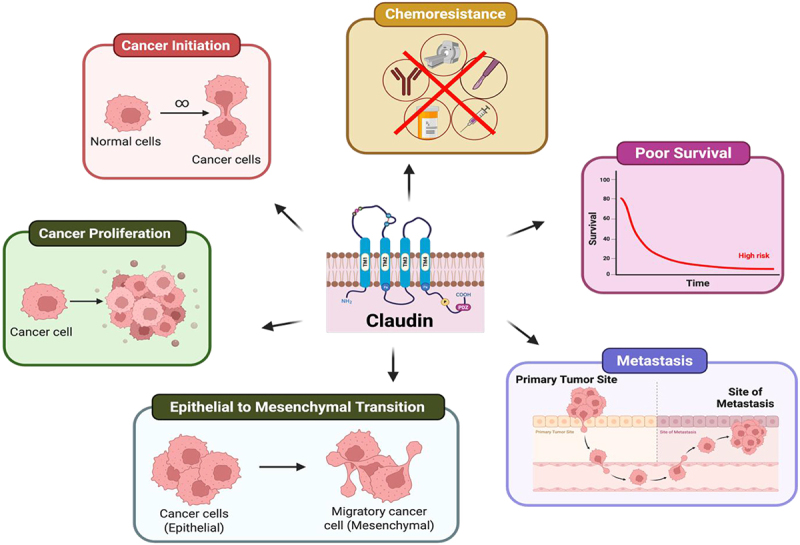
Schematic representation of the role and involvement of claudins in ovarian cancer. Created in https://BioRender.com.

CLDN18.2 (Claudin 18 splice variant 2), one of two isoforms of CLDN18, normally found on the gastric epithelium, is observed to be ectopically expressed in OC.^119^ Wagner et al. investigated the expression of CLDN18.2 in tubo-ovarian carcinoma (TOC) ;(*n* = 536), their corresponding metastatic tissues (*n* = 385), and in 93 metastases without primary tumors via IHC staining. They found that the CLDN18.2 expression was mostly seen and restricted to the mucinous subtype, and therefore concluded this claudin to be a promising target for personalized therapy in patients with advanced mucinous TOC.^120^ Claudin18.2 is also highly expressed in primary ovarian mucinous carcinoma (i) and metastatic gastrointestinal mucinous carcinoma (MGMC) derived from upper gastrointestinal tract primary tumours and is a potential candidate for therapeutic targeting in these cancers.^121^ Role and involvement of claudins and their functional significance in OC is illustrated in Figure 2.

Claudins, mainly -1,-3,-4,-6,-7,-16 and -18.2 are overexpressed in ovarian cancer. Overexpression of these claudins mediates induction of several molecular signaling pathways such as ERK/MAPK, thereby modulating epithelial-mesenchymal transition (EMT), which promotes invasion, migration, and chemotherapy resistance.

## Claudins as prognostic and diagnostic biomarkers in OC

As explained above, most of the claudins are reported to be overexpressed in OC, suggesting that they may have a tumor-promoting role.^68^ They offer a noninvasive screening possibility as of their detectable expression in blood exosomes.^122^ This section covers the findings that suggest the role and importance of claudins in OC.

Reports suggest that OC patients’ exosomes with CLDN4 were present in almost 51% of plasma samples, whereas it was only 2% in normal healthy individuals. This group suggests that identification of claudin in blood-derived exosomes could revolutionize early detection of OC.^123^ Hegab et al. suggest that high claudin expression, especially CLDN4, is correlated with advanced tumor stage, lymph node metastasis, and chemoresistance. Moreover, the study reports that elevated CLDN4 expression could be a predictive marker for poor chemotherapeutic response, poor survival, and is correlated to residual tumor post-surgery in advanced OC.^86^ Another study shows that CLDN5 overexpression can be predictive of shorter cancer-specific (*p* = 0.032) as well as OS (*p* = 0.026), with a 5-year survival rate of 25–30% in CLDN5-positive patients. In CLDN5 negative patients, the OS was found to be 60%.^97^ When the expression is found to be stronger in high-grade and late-stage cancers, it could be correlated to the aggressiveness of the disease.^97^ CLDN 7 is found to modulate EMT and Erk pathways, thereby influencing cellular metastasis and chemotherapy resistance in OC cells.^124^ This study, which included 95 ovarian tissue samples, including normal and various cancer subtypes, reported that inhibition of CLDN7 using SiRNA resulted in dysregulation of many genes involved in cell growth, cell division, and cell death, but paradoxically reduced migration mediated by Erk signaling.^121^ This suggests that CLDN4, CLDN5, and CLDN7 are potential vigorous biomarkers with unique roles in chemoresistance (CLDN4), survival (CLDN5), and metastasis (CLDN7).

Claudin expression profile is found to be varying across different histological subtypes of OC.^68^ This subtype-specific expression patterns of claudins, such as CLDN4 in high-grade serous tumors and CLDN7 in all epithelial subtypes, highlight their potential as prognostic tools and therapeutic targets. In serous carcinomas, overexpression of claudins is associated with advanced stage and chemotherapy resistance.^86^ In endometrioid and mucinous subtypes, CLDN7 expression is found to be higher and is correlated to invasion but not cellular migration.^109^

The potential use of claudins in combination with other existing biomarkers in improving diagnostic precision has also been implicated. Reports suggest that claudins (especially CLDN4) in combination with existing screening methods/markers such as CA125 could help elevate diagnostic accuracy.^123^ CLDN3 in combination with CA125 and MUC1 is reported to detect more than 99% of EOCs in tissue samples despite the heterogeneity of the disease.^68^ This further elevates the importance of claudins and their potential to transform OC management.

MYCN is known to regulate genes involved in cell proliferation, differentiation, and survival.^125^ Recent transcriptomic and genomic profiling studies have identified MYCN amplification and overexpression as hallmarks of the C5 molecular subtype of high-grade serous ovarian carcinoma, which is characterized by stemness, mesenchymal features, and poor clinical outcomes.^126^ These tumors exhibit reduced epithelial differentiation, reminiscent of a “claudinlow” phenotype, suggesting a broader rewiring of tight junction protein expression, including claudins, in this aggressive subtype.^127^

Although no studies have yet directly linked MYCN to the transcriptional regulation of claudin genes in ovarian cancer, the co-occurrence of MYCN-driven dedifferentiation and a claudin-low state implies a functional relationship. The C5 subtype displays both elevated MYCN and reduced claudin expression, consistent with disruption of epithelial tight junction.^126^

Although early-phase clinical trials of claudin-targeting therapeutic agents show promise in OC, further research into isoform-specific strategies and combination approaches is crucial to translate preclinical success into durable clinical responses, especially for chemo-resistant tumors. Harnessing claudins’ dual diagnostic and therapeutic utility could revolutionize OC management by addressing unmet needs in treatment resistance and metastatic progression.

## Therapeutic targeting of claudins in OC

Due to their heterogeneous expression patterns across various OC histotypes, roles in tumor aggression, metastasis, and chemoresistance, several claudin members have emerged as potential therapeutic targets in OC. Claudins, due to their extra-junctional mis-localization in tumor cells, have become an interesting candidate for drug delivery targets with minimal adverse effects. In that, several approaches have been explored, including Clostridium perfringens enterotoxins (CPEs), monoclonal antibodies (mAbs), CPE-binding domains (C-CPEs), mAb – drug conjugates (ADC), bispecific T-cell engagers (BiTEs), and chimeric antigen receptor (CAR)-T cells.^128^ In this section, we will summarize the findings of studies that discuss the current approaches primarily used for targeting claudins in OC, as well as the findings of ongoing early-phase clinical trials indicating the potential significance of claudin-targeted agents in OC diagnosis, treatment, and maintenance. Table 2 summarizes the claudin targeted therapeutic strategies in OC treatment.

**Table 2.** Claudin-targeted therapeutic strategies in OC treatment. Preclinical studies have shown that targeting claudins with CPE-based cell lysis, CAR-T cells, mAbs, and antibody-drug conjugates enhances tumor-specific cytotoxicity and can help overcome chemoresistance.

Claudin	Therapeutic Potential	Targeting agent(s)	Preclinical/Clinical Status	Advantages	Reference
Claudin-1	Targeting with a monoclonal antibody	6F6 (mAb)	Preclinical	Inhibits invasion and cancer growth	^132^
Claudin-3	Target for CPE-based agents, mAbs, ADCs	cKM3907 (mAb) h4G3 (mAb) human IgG1 anti-claudin3 antibody scFvH6 (mAb) FNR648 with CLDN3-specific antibody conjugate (ADC)	Preclinical, early clinical	High specificity, cytolytic effect, targeted delivery,	^131,136^
Claudin-4	Target for CPE-based agents, mAbs	KM3900 (mAb) cKM3907 (mAb) KM3934 (mAb)	Preclinical, early clinical	High specificity, cytolytic effect targeted delivery,	^97,134,135^
Claudin-6	Targeting with mAbs, ADCs, CART, BiTE	CLDN6-23-ADC (ADC) TORL-1–23 (ADC) AMG 794 (BiTE) BNT142 RNA-LNP (BiTE) BNT211-01 (CART) BNT211 (CARVac)	Clinical trial	Successful elimination of subcutaneous human xenograft tumors, T cell-mediated cell death	^99,138,139,141,142,144^

## Clostridium perfringens enterotoxin (CPE)

Clostridium perfringens enterotoxin (CPE) and its C-terminus domain (C-CPE) recognizes and binds to specific amino acid sequences in the extracellular loops of claudins −3, −4 and −6 leading to their disruption, damaging plasma membrane and has already been shown to resensitize resistant tumors^87^ ultimately leading to cell death via calcium influx.^93,129,130^ This feature of CPE is exploited to enhance drug delivery to cancer cells. C-terminal fragment of CPE is shown to improve the efficacy of chemotherapy in OC,^131^ and has also been researched as a possible drug carrier.^132^ To analyze the optimal conditions for CPE as an anti-cancer drug for treating OC *in vitro* and *in vivo*, Tanaka et al. cultured OC cells wherein they found that the cells at low density had higher sensitivity to CPE and CLDN4 at TJs were less-reachable to CPE.^133^ This provides important information indicating that the accessibility of CLDN4 at the cell surface is crucial for CPE cytotoxicity. Santin et al. report that CLDN3 and 4 overexpressing primary ovarian tumors, irrespective of their sensitivity to therapeutic drugs, were dead within 24 hr upon CPE exposure of 3.3 microg/mL in vitro. Moreover, they also showed that multiple intra-peritoneal administrations of sublethal CPE doses significantly inhibited tumor growth in SCID mice explanted with OVA-1 cells.^90^ They suggest CPE-based therapy as a novel strategy for OC treatment. Though this is a promising method, lack of tumor ;specificity, however ;, limits its clinical use.^119^ Cocco et al. reported the use of the CPE peptide as a drug delivery agent as well as an imaging agent in OC.^134^ In that, a fluorescein isothiocyanate (FITC)-conjugated C-CPE peptide has been engineered to selectively bind OC cells expressing claudins 3 and 4, demonstrating specificity in vitro and in vivo.^134^

## Monoclonal antibodies (mAb) targeting claudins

Antibodies targeting claudins provide a beneficial treatment option for the specific targeting of claudin-overexpressing malignancies.^135^ This could also limit chemotherapy-associated toxicities due to the usage of reduced doses, ensuring similar therapeutic effects.^119^ The antibody is found to effectively decrease colony formation as well as the number of colonies in various CLDN1-overexpressing OC cell lines, including SKOV3 and IGROV1. Reports suggest that 6F6 also significantly inhibited tumor growth and liver metastasis in a colorectal cancer xenograft model.^136^ KM3900 (IgG2a) is an antibody generated by Suzuki et al. that targets CLDN4. They targeted CLDN4 with this antibody, which ultimately resulted in dose-dependent, antibody-dependent cellular cytotoxicity (ADCC) and complement-dependent cytotoxicity (CDC) in vitro, as well as in vivo tumor growth inhibition in mice models.^97^ Recently, murine-human chimeric antibodies against CLDN4 and chimeric dual targeting monoclonal antibodies against CLDN4 and CLDN3 have been developed and tested for their antitumor characteristics. They were found to significantly create a dose-dependent effect on OC cells.^119^ The use of antibody cKM3907, engineered with an IgG1 Fc domain, showed a ;complement-dependent cytotoxicity (CDC) ;that resulted in significant suppression of tumor formation ;in SCID mice inoculated with CLDN3- or CLDN4-transfected Chinese hamster ovary (CHO) cells. Moreover, a likewise antitumor efficacy was also noticed in MCAS cells, a human OC line co-expressing CLDN3 and CLDN4, where cKM3907 administration ;inhibited measurable tumor growth.^137^ Reports suggest that humanized antibodies KM3934, targeting OC cells when co-cultured with PBMCs and xi-5D12, effectively against human/mouse CLDN4-expressing cells, notably exhibited ADCC by binding to CLDN4‘s extracellular domain 2.^138,139^ Yang et al. developed human mAb (h4G3) against CLDN3 through scFv phage display using CLDN3-expressing CHO-K1 cells and CLDN3-embedded lipoparticles. This antibody showed high specificity to CLDN3 and a CLDN3-dependent ADCC activity in tumors.^140^

## Antibody-drug conjugates (ADCs)

Antibody-drug conjugates are also being developed for clinical use. In that, Romani et al. proposed a novel human IgG1 anti-claudin3 antibody that can specifically recognize aberrantly localized CLDN3 in OC cells and that is suitable for selective drug delivery.^135^ This group engineered a fully human anti-CLDN3 IgG1 antibody (IgGH6) by fusing the human IgG1 Fc-domain to the anti-CLDN3 scFvH6. This antibody detects and binds to a specific epitope found in CLDN3’s minor extracellular loop, that are observed and accessible only in tumor cells lacking organized tight junctions.^135^ Oh et al. successfully conjugated a radioisotope and a fluorescent protein (FNR648) with CLDN3-specific antibodies that showed a 2.5-fold higher tumor uptake (20.4 ± 7.4% ID/g) at 24 h post-injection in OVCAR3 tumor xenografted mice.^141^ Antibody-drug conjugate CLDN6-23-ADC (anti-CLDN6), showcased a significant reduction in the tumor volume in an ovarian PDX model and could be a promising personalized therapeutic strategy currently in phase I clinical trial for the treatment of CLDN6 overexpressing cancers.^142^ Very recently, it has been demonstrated that TORL-1–23, an ADC against CLDN6, is very well tolerated and has proven favorable safety/efficacy in patients with heavily pretreated, CLDN6-positive advanced cancers, including platinum-resistant OC, according to the phase 1 TORL-123–001 clinical trial (NCT05103683) findings.^143^ An innovative bispecific ADC targeting different cludins simultaneously can, however, enhance tumor heterogeneity.

## Bi-specific T cell engagers (BiTes)

BiTE refers to bi-specific antibody constructs containing single-chain variable domain fragments that bridge cytotoxic T cells to tumor cells, initiating target-dependent polyclonal T-cell activation and proliferation, leading to cell death.^144^ AMG 794 is a half-life extended BiTE targeting CLDN6. In vitro studies have proven that AMG 794 induces human T cells-mediated cell death of CLDN6-expressing cancer cells. Reports suggest that weekly dosing of AMG 794 significantly inhibited the growth of established lung and ovarian xenograft tumors in immunocompromised mice injected with human T cells.^145^ BNT142, a lipid nanoparticle (LNP)-formulated RNA (RNA-LNP), encodes another bispecific antibody named RiboMab02.1, which binds to the T cell marker CD3 and CLDN6.^146^ Weekly injections of mice with BNT142 RNA-LNP (0.1- to 1-μg) is found to significantly eliminate CLDN6-positive subcutaneous human xenograft tumors.^146^

## Chimeric antigen receptor T-cell (CAR-T) therapies

**Figure 3.**
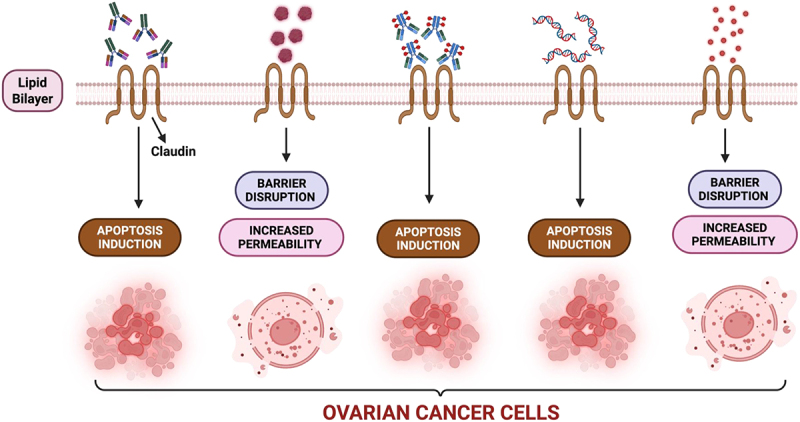
Emerging therapeutic strategies targeting claudins in ovarian cancer.

Chimeric antigen receptor T-cell (CAR-T) therapies, primarily effective in hematologic malignancies, have opened doors for exploring their potential in treating solid tumors, such as OC.^147^ In OC, there is an ongoing phase trial evaluating the efficacy of CART cells, BNT211-01, that specifically target CLDN6 cells^148^. ;RNA vaccines conjugated with CART, CARVac (e.g., BioNTech’s CARVac) ;could elevate the efficacy of CART by boosting CLDN6-specific immunity.^99^ BNT211 is CARVac that showed a 33% objective response rate in relapsed CLDN6-positive cancers, including OC.^148^ Figure 3 depicts the major therapeutic strategies currently being explored to target claudin family proteins in OC.

This schematic representation illustrates the major therapeutic approaches under investigation for targeting claudin family proteins in ovarian cancer. Claudins, integral components of tight junctions, are frequently dysregulated in ovarian cancer and present as promising therapeutic targets due to their cell-surface localization and subtype-specific overexpression. The therapeutic strategies include:

**Monoclonal antibodies (mAbs)**: Target extracellular loops of claudin proteins (e.g., CLDN3, CLDN4), leading to immune-mediated cytotoxicity and induction of apoptosis in claudin-overexpressing ovarian cancer cells.

**Clostridium perfringens enterotoxin (CPE)**: A natural ligand for CLDN3/CLDN4, CPE and its modified derivatives bind selectively to these claudins, disrupting tight junction integrity and causing selective cytolysis or increased paracellular permeability.

**Antibody – Drug Conjugates (ADCs)**: Engineered to combine claudin-specific antibodies with cytotoxic payloads, ADCs enable targeted intracellular drug delivery, resulting in apoptosis and reduced systemic toxicity.

**siRNA-based therapies**: Small interfering RNAs designed to knockdown expression of oncogenic claudins, thereby restoring epithelial integrity and triggering apoptosis or growth inhibition.

**Small-molecule inhibitors**: Compounds that interfere with claudin expression or localization, modulate tight junction signaling, or enhance paracellular permeability to potentiate drug delivery.

Downstream effects of these interventions include the induction of apoptosis, disruption of the barrier, and modulation of the immune system, offering multiple avenues to suppress tumor growth and enhance therapeutic response. These strategies are at various stages of development, from preclinical testing to early-phase clinical trials, and represent a frontier in precision therapy for claudin-overexpressing ovarian cancer. Created in https://BioRender.com.

## Challenges and future directions

Studies prove that claudins play a crucial role in tumorigenesis and can be a potential therapeutic target for many solid as well as hematological malignancies.^149^ Moreover, as the fundamental components of tight junctions, they could serve as promising targets for regulating tissue permeability to deliver drugs in various disease treatments.^150^ However, despite their relevance, several obstacles impede development in claudin research. Until now, 27 members of the claudin family have been identified,^28^ most of which share similar structural and functional characteristics that might complicate the interpretation of gene knockout or knockdown studies. A major obstacle is the systemic toxicity of claudin targeted therapies, as claudins are widely expressed in tissues. Claudin-targeted therapies, predominantly mAbs and ADCs, hold substantial potential but face challenges associated with target accessibility, pharmacokinetics, and systemic toxicity. ADCs are observed to cause systemic toxicity due to their nonspecific uptake as well as impartial release resulting in hematologic, hepatic, and gastrointestinal side effects.^151^ Therapies with claudin-targeting antibodies may induce immune responses, thereby generating infusion reactions or cytokine release syndrome.^119^ One of the major disadvantages of targeted therapy is the difficulties associated with accessibility. For example, claudin 18.2 is deeply localized in a way that the epitopes are largely inaccessible to the antibodies, thereby reducing their effects.^152^ Moreover, the presence of multiple isoforms within the claudin family across different tissues poses a challenge to achieving consistent effects, due to reduced specificity.^119^ Ensuring isoform specificity, optimizing drug delivery at the target site, and handling adverse effects are enduring areas of clinical development in this scenario. Though disruption of tight junction promotes drug delivery/uptake, it can favor tumor promotion by increasing nutrient absorption.^153^ Monoclonal antibodies, antibody drug conjugates, and various other therapeutic interventions have been extensively tested and verified in basic and preclinical research. However, the clinical development of these therapeutic agents has not progressed significantly to clinical application levels.

Furthermore, certain pharmacokinetic limitations, including difficulties with antibody/ADC penetration due to the huge size of solid tumors and the ECM layer, could also potentially reduce the efficacy of therapy.^119^ Variations in the expression profile of claudins depending on the tumor types, and patients further complicate uniform drug delivery and efficacy.^154^ Despite being overexpressed in OC, claudins are not entirely tumor-specific; their presence in normal cells can lead to therapeutic agents being absorbed to non-tumor sites, reducing the available drug concentration and altering their pharmacokinetic parameters, including distribution pattern and clearance from the system.^27,68^ Moreover, repeated dosing of antibodies can induce the production of anti-drug antibodies (ADAs) that can neutralize them, favoring their clearance from the site.^119^

Besides, mouse models from in vivo studies, though showed promising effects in claudin-based disorders, they do not fully reiterate human phenotypes, emphasizing the difficulties of animal models mimicking human pathophysiology.^155^

Though targeted therapies hold significant promise for the treatment of ovarian cancer due to the frequent overexpression of claudins in OC cells; as discussed above, they possess major pharmacokinetic limitations, such as poor tumor penetration, issues related to differential expression of claudins across tumor sites, and their presence in normal tissues causing a ‘sink effect,’ that can confiscate the drug thereby reducing its obtainability at the tumor site. These aspects, besides several others, such as clearance rates and possible systemic toxicity due to binding at non-tumor sites, further obscure the clinical relevance of these agents. Overcoming these challenges, however, requires highly specific antibodies and ADCs, as well as more advanced drug delivery systems, such as nanoparticles. Combination therapies could be a better option, potentially improving the effect, and improvements in techniques could reduce toxicity and safety concerns. A better approach would be the use of AI and machine learning in patient selection and a real-time monitoring of treatment response. Together, all these revolutions can potentially renovate Claudin-targeted therapies to a better and safer choice for OC patients, resulting in improved clinical outcomes.

## Conclusion

Dysregulated expression of various claudin members has been observed in OC. Although the current understanding of the exact role and involvement of claudins in OC is limited, experimental evidence suggests that claudins can be exploited as a molecular prognostic marker, diagnostic marker, and potential therapeutic target for OC treatment. Their role in tumorigenesis, cellular invasion, metastasis, and therapy resistance positions them as critical mediators of treatment failure. Their potential characteristic being a target of CPE-based treatment (especially CLDN3 and CLDN4) has demonstrated a promising value for intracellular delivery of therapeutic drugs. Claudins represent a transformative frontier in OC therapy, with CLDN6 and CLDN18.2 leading clinical translation. Addressing tumor heterogeneity and chemoresistance will be critical for realizing their full potential. Preclinical studies have demonstrated that targeting claudins using CPE-based cytolysis, CAR-T cells, monoclonal antibodies, and antibody drug conjugates has proven improved tumor-specific cytotoxicity and helps overcome chemoresistance, as suggested by many studies involving xenograft models. However, a thorough understanding of claudins in OC can establish a basis for enhancing diagnostic, predictive, and therapeutic approaches that may result in improved therapy outcomes.
